# Serum midkine as non-invasive biomarker for detection and prognosis of non-small cell lung cancer

**DOI:** 10.1038/s41598-021-94272-8

**Published:** 2021-07-16

**Authors:** Louisa Stern, Erik Mueller, Eugen Bellon, Matthias Reeh, Rainer Grotelueschen, Cenap Guengoer, Nathaniel Melling, Mara Goetz, Daniel R. Perez, Jakob R. Izbicki, Tamina Rawnaq-Möllers, Tarik Ghadban

**Affiliations:** Department of General, Visceral and Thoracic Surgery, University Medical Center Hamburg-Eppendorf, Martinistrasse 52, 20246 Hamburg, Germany

**Keywords:** Non-small-cell lung cancer, Cancer screening, Tumour biomarkers

## Abstract

Lung cancer continues to be the leading cause for cancer-related deaths in men and women worldwide. Sufficient screening tools enabling early diagnosis are essential to improve patient outcomes. The aim of this study was to evaluate serum midkine (S-MK) both as a diagnostic and prognostic biomarker in non-small cell lung cancer (NSCLC). This single-center analysis included 59 NSCLC patients counting 30 squamous cell cancers and 29 adenocarcinomas. Preoperative S-MK concentration was determined using ELISA. Patients were followed up to five years. S-MK was found to be significantly overexpressed in patients with NSCLC compared to healthy controls (p < 0.001). The discriminative power of S-MK to differentiate NSCLC subjects from controls was fairly high with an area under the receiver operating characteristic curve of 0.83 (p < 0.001). Optimal sensitivity of 92% and reasonable specificity of 68% was reached at a threshold of 416 pg/ml S-MK. Patients with high S-MK concentration showed a significantly shorter overall survival compared to patients with low S-MK expression (p < 0.05). In conclusion, S-MK is overexpressed in patients with NSCLC and serves as an independent prognostic factor for overall survival. S-MK may thus be considered as an additional non-invasive biomarker not only for NSCLC screening but also for outcome prediction.

## Introduction

Lung cancer remains by far the leading cause for cancer-related mortality for both women and men worldwide^1^. Based on the histopathological type lung cancer is divided into small cell lung cancer (SCLC) and non-SCLC (NSCLC). NSCLC accounts for approximately 85% of all cases^2^ and can be further subdivided into the two most common types: adenocarcinoma and squamous cell cancer. For NSCLC the 5-year survival rate is approximately 17%^3^. Survival prognosis thereby strongly depends on the cancer stage since only early tumor stages can be treated with curative intent^3^. Due to late-onset of symptoms, aggressive tumor behaviour and the lack of sufficient screening tools most patients do not get diagnosed before advanced tumor stages are reached^4,5^. Hence, advances in screening technologies are essential in order to facilitate early detection and thereby improve the outcome of NSCLC patients. In addition, the management of locally advanced NSCLC is still under debate. Predictive biomarkers can be used to identify tumors that are suitable for targeted therapies, this way enabling an individualized treatment approach.

Serum-Midkine (S-MK) has already been proposed as a potential biomarker for different tumor entities including gastrointestinal and lung cancer^6^. MK is a pleiotropic growth binding protein initially found to be highly up-regulated during embryogenesis thereby playing a key role in neuronal differentiation^7,8^. From birth onwards, it usually remains downregulated to a low background expression level in healthy adults^6,9^. Recent studies have reported elevated expression levels of S-MK in various cancer types including hepatocecullar and lung cancer^10^. More importantly, numerous reports have shown that MK overexpression is an indicator of impaired prognosis also for NSCLC patients^11^. Furthermore, MK has been found to decrease after surgical tumor removal and rebound prior to tumor recurrence in the case of breast and hepatocellular cancer^12,13^. In recent years, a considerable number of analyses have provided evidence for a functional role of MK in molecular tumor biology. In vitro studies have shown that MK exhibits antiapoptotic and angiogenic activities leading to enhanced cell proliferation in lung cancer amongst other tumor entities. Moreover, MK was shown to promote chemoresistance through Notch signalling^14^. Since MK is a soluble, secreted cytokine, serum levels strongly correspond with protein expression levels in tumors^6^. Therefore, MK expression can be easily quantified in peripheral blood samples, making it a non-invasive and inexpensive diagnostic tool, feasible in the setting of the standard preoperative assessment. The aim of this study was to further evaluate the clinical significance of MK in NSCLC by studying both its diagnostic and prognostic value as a potential serum biomarker for NSCLC.

## Materials and methods

### Patients and study design

For this study, NSCLC patients who underwent resection with curative intention between 1994 and 2011 at the Department of General, Visceral and Thoracic Surgery at the University Medical Centre Hamburg-Eppendorf were followed. Blood samples were taken in the setting of preoperative assessment and analysed retrospectively. The histopathological diagnosis was made by a pathological specialist at the Pathological Institute at the University Medical Centre Hamburg-Eppendorf. Histological classification of the tumor was done according to the sixth edition of the American Joint Committee on Cancer (AJCC). Only patients with histologically confirmed NSCLC and tumor-free resection margins (R0) were finally included in the study. These criteria were applicable to a total of 59 NSCLC patients. The NSCLC group was comprised of 48 male and 11 female patients with a median age of 65 years ranging from 37 to 82 years. Squamous cell cancer (n = 30) and adenocarcinoma (n = 29) were equally distributed. All patients were treated according to the German national guidelines for NSCLC. Patients were followed up to five years. This included medical history, physical examination, and a CT-scan every three months for the first two years followed by every six months for another three years. All data including sex, age, tumor stage, metastasis, recurrence and overall survival were obtained prospectively.

75 blood bank donors served as healthy control for the study. The median age was 49 years. Patients did not exhibit any known relevant medical conditions, especially no malignant or chronic inflammatory disease.

Written informed consent was obtained from all patients for using serum samples and tissue samples. All aspects of the study were approved by the ethics committee of the University of Hamburg, Germany (PV3548) and were carried out in accordance with the approved guidelines.

### Enzyme-linked immunosorbent assay for human midkine

All blood samples were taken preoperatively and stored at − 80 °C until measurements were performed. S-MK levels were measured using a commercially available enzyme-linked immunosorbent assay (ELISA) (BioVendor, Heidelberg, Germany) according to the manufacturer’s instructions. First, NSCLC patients’ serum was added to microtiter wells precoated with a polyclonal primary rabbit antibody raised against human MK. Following an incubation period of 1 h, two consecutive washing steps were performed to remove all unbound primary antibody. Next, a biotin-labelled polyclonal anti-human MK secondary antibody was added and incubated for another hour. This was followed by several washing steps. Finally, a streptavidin–horseradish peroxidase conjugate was added to convert the substrate H_2_O_2_-tetramethylbenzidine. The reaction was stopped by the addition of an acid solution. Finally, absorbance was measured photometrically at 450 nm (OD450) using a microplate reader (Dynatech MR 500). Human recombinant MK (Chemicon International, Temecula, CA) was used as a positive control. A calibration curve plotted with a set of standard samples of a known concentration was used to determine the exact MK concentration. MK concentration is expressed in pg/ml.

### Statistical analysis

SPSS for Macintosh (Version 26.0.0.0 SPSS Inc., Chicago, IL) was used for statistical analysis. Equal variance was assessed using Levene’s test. Statistical significance was evaluated by a parametric t-test and one-way ANOVA Kruskal–Wallis test. Receiver operating characteristic (ROC) curve analysis was used to assess the discriminatory power and to determine the optimal cut-off value. Survival analysis of the patients was plotted by the Kaplan–Meier method and analyzed using the log rank test. Independent risk factors were assessed by multivariate analysis using cox regression. Results are presented as median survival with 95% confidence interval (95% CI) and the number of patients at risk. In case the median survival was not reached, mean values are presented and specifically indicated. The overall survival was computed as the time period from the date of surgery to either the date of death or last follow-up, whichever occurred first. The disease-free survival was defined as the time period from the date of surgery to the date of recurrence, last follow-up or date of death, whichever occurred first. Patients alive without recurrence at the follow-up date were censored^15^. A p-value < 0.05 was considered as statistically significant.

## Results

**Figure 1:**
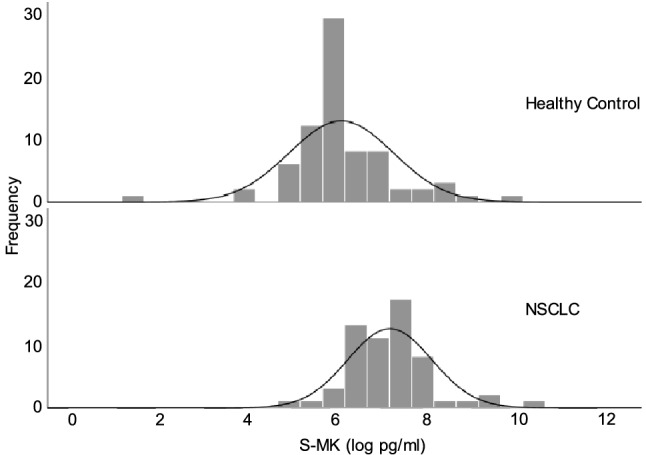
Frequency distribution of S-MK concentration in healthy adults and patients with NSCLC. Abbreviations: *S-MK* serum-midkine, *NSCLC* non-small lung cancer, *pg* picogram, *ml* milliliter. Data displayed as a log-scaled histogram overlaid with a normal distribution curve. Levene’s test was used to confirm the equality of variances (p = 0.58).

MK expression was determined preoperatively in 59 NSCLC patients and compared to MK expression in 75 healthy individuals by measuring S-MK using ELISA. The frequency distribution of S-MK concentration was plotted on a log-scaled histogram as illustrated in Fig. 1. A normal distribution with equal variance was found in both groups (Levene’s test p = 0.58).

**Figure 2:**
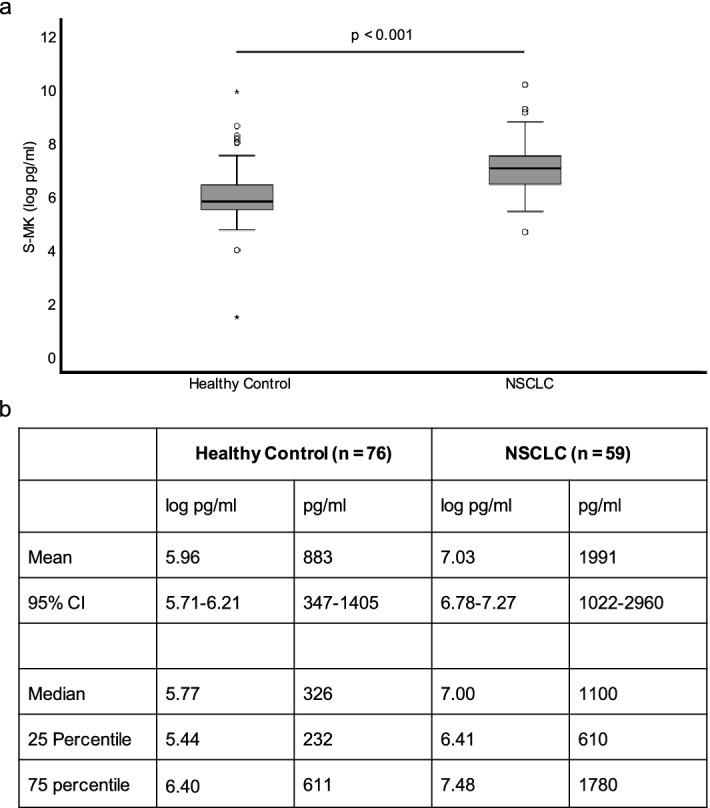
S-MK expression is upregulated in patients with NSCLC compared to healthy controls. Abbreviations: *S-MK* serum-midkine, *NSCLC* non-small lung cancer, *pg* picogram, *ml* milliliter, *CI* confidence interval. (**a**) Data are shown in a box-and-whisker plot (median, 25th and 75th percentile, range, extreme values outside the range). (**b**) Data are expressed as means +/− 95% CI and medians +/− interquartile ranges. Statistical significance was determined using an unpaired t-test, p < 0.001.

In patients with NSCLC median expression levels of S-MK were more than three times higher (median 1100 pg/ml, 25–75 percentile 610–1780 pg/ml) than the S-MK expression levels in the healthy control group (334 pg/ml, 25–75 percentile 232–611 pg/ml). The mean difference in MK concentration accounted for 1.08 log pg/ml (95% CI 1.44–0.72) which corresponds to an actual value of 1108 pg/ml and was statistically significant (p < 0.001) (Fig. 2).

**Figure 3:**
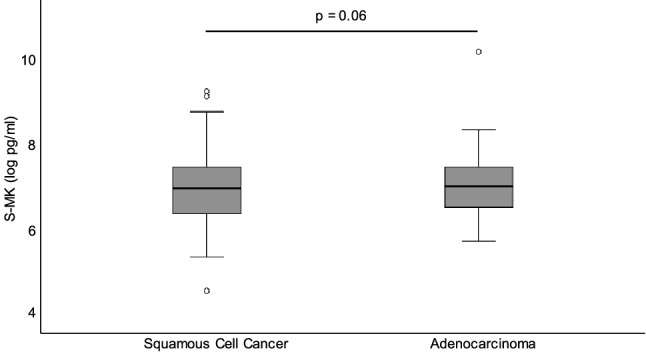
No difference in S-MK concentration between adenocarcinoma and squamous cell cancer. Abbreviations: *S-MK* serum-midkine, *pg* picogram, *ml* milliliter. S-MK concentration was compared between NSCLC patients with adenocarcinoma and squamous cell cancer. Data are shown in a box-and-whisker plot (median, 25th and 75th percentile, range, extreme values outside the range). Statistical significance was evaluated using unpaired t-test, p = 0.58.

Looking at the subentities of NSCLC, there was no significant difference in S-MK concentration between adenocarcinoma and squamous cell cancer as shown in Fig. 3.

**Figure 4:**
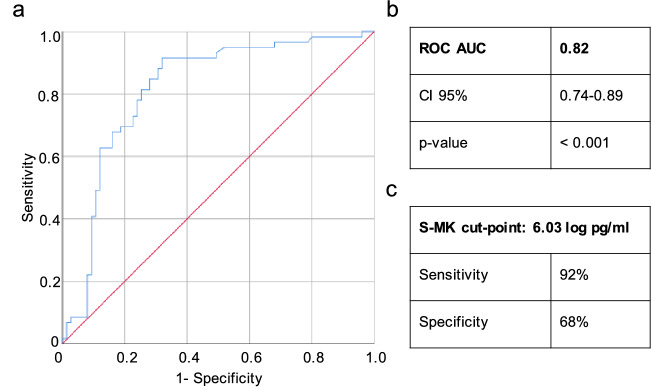
High diagnostic power of S-MK concentration to discriminate NSCLC from healthy controls. Abbreviations: *S-MK* serum-midkine, *NSCLC* non-small lung cancer, *pg* picogram, *ml* milliliter, *CI* confidence interval. (**a**) The blue solid line represents an estimated ROC curve for distinguishing NSCLC patients from healthy individuals based on the S-MK concentration. Sensitivity (true positive rate) is plotted versus 1-specificity (false positive rate) for all possible values of S-MK concentration. The red line reflects the ROC curve for random guessing. (**b**), (**c**) The calculation is based on the retrospective analysis of 59 NSCLC patients and 73 healthy control individuals. AUC = 0.82 (CI 95% 0.741–0.892; p < 0.001).

After having confirmed MK overexpression in NSCLC patients, the next step was to evaluate its predictive power as a screening tool to distinguish malignant NSCLC patients from healthy adults in more detail. For this purpose, a ROC curve was calculated. As shown in Fig. 4 the sensitivity (true positive rate) versus one minus specificity (false positive rate) was plotted across the whole range of all possible S-MK thresholds^16^. The area under the curve (AUC) thereby reflects the degree of separability between normal and abnormal values. The larger the AUC the greater the certainty of NSCLC prediction based on the S-MK concentration. The AUC was found to be 0.82 (p < 0.001) suggesting a relatively high diagnostic power (Fig. 4)^16^.

Once a decent separability power had been verified, the ROC-curve was used to determine the optimal S-MK threshold to predict NSCLC. The latter is the value whose sensitivity (true positive) and 1- specificity (false positive) are the closest to the value of the area under the ROC curve thereby classifying most of the individuals correctly as malignant or benign^16^. In the case of a screening test an exquisitely high sensitivity and reasonable specificity is required^16^. Based on our data an optimal cut-off value for S-MK to detect NSCLC patients was found to be at 6.03 log pg/ml S-MK which means an absolute value of 416 pg/ml. The corresponding sensitivity and specificity are 92% and 68%, respectively.

**Figure 5:**
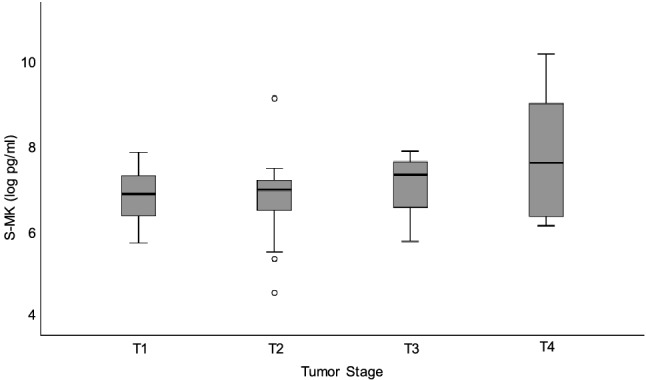
Trend towards increasing S-MK levels with NSCLC tumor stage progression. Abbreviations: *S-MK* serum-midkine, *NSCLC* non-small lung cancer, *pg* picogram, *ml* milliliter. Data shown in box-and-whisker plot (median, 25th and 75th percentile, range, extreme values outside the range). The level of statistical significance was determined using Kruskal–Wallis one-way ANOVA (p = 0.42).

Next, the distribution of MK expression within the group of NSCLC patients was analyzed. S-MK levels were correlated with clinical and pathological parameters in order to evaluate its clinical significance in NSCLC. Due to a normally distributed set of data Kruskal–Wallis one-way ANOVA was used to test for statistical significance. All clinicopathological parameters recorded are summarized in Table 1. There was no statistically significant correlation between the above-mentioned parameters and the S-MK concentration. However, there was a clear trend towards gradually rising S-MK expression levels with advanced tumor stages as indicated in Fig. 5 (p = 0.42).

**Table 1:** Correlation of clinicopathological parameters with S-MK concentration.

Parameter	Number of patients	SMK concentration Median (log pg/ml)	SMK concentration Median (pg/ml)	Kruskal–Wallis p-value
**Gender**				
Male	48	6.99	1086	0.71
Female	11	7.21	1353	
**Smoker**				
Non-smoker	5	6.91	1002	0.97
Smoker	54	7.02	1119	
**Age**				
< 60	18	6.973	1070	0.54
≥ 60	41	7.00	1100	
**Histological subtype**				
Squamous cell cancer	30	6.99	1086	0.68
Adenocarcinoma	29	7.04	1141	
Tumor stage*				
T1	17	6.91	1002	0.42
T2	23	6.7	812	
T3	11	7.4	1636	
T4	8	7.6	1998	
Lymph node status*				
N0	23	6.91	1002	0.50
N1	26	7.18	1313	
N2	10	6.92	1012	
Distant metastasis*				
M0	54	7.07	1176	0.50
M1	5	6.40	602	
UICC*				
I	17	6.86	953	0.05
II	19	7.26	1422	
III	17	7.19	1326	
IV	6	6.44	626	
Grading*				
G1	3	6.86	953	0.70
G2	22	6.91	1002	
G2	34	7.07	1176	
**Local recurrence**				
No	51	6.91	1002	0.06
Yes	8	7.46	1737	
**Distant recurrence**				
No	27	6.92	1012	0.24
Yes	32	7.02	1119	

*S-MK* serum-midkine, *UICC* Union for International Cancer Control.

*Pathological evaluation according to the guidelines of the Union for International Cancer Control 2017.

**Figure 6:**
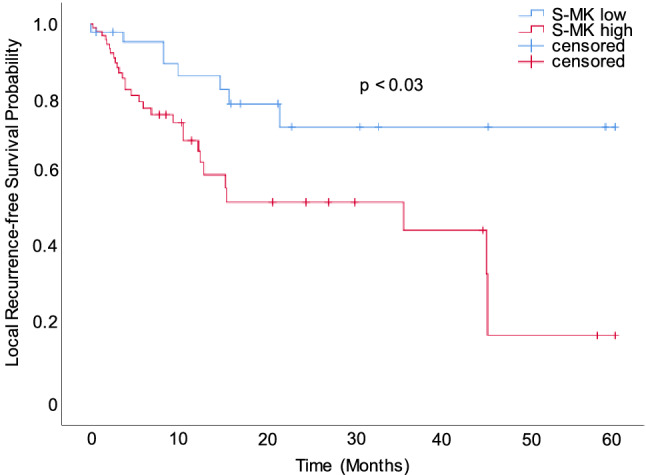
Prolonged local recurrence-free survival time in NSCLC patients with low S-MK expression compared to high S-MK expression. Abbreviations: *S-MK* serum-midkine, *NSCLC* non-small lung cancer. Kaplan–Meier curve comparing local recurrence-free survival of NSCLC patients with low (S-MK < 6.71 log pg/ml; n = 22) and high s-MK levels (S-MK ≥ 6.71 log pg/ml; n = 37). Patients were followed up for 5-years post-surgery. Statistical difference was determined using the log-rank test (p < 0.03).

Moreover, elevated preoperative S-MK levels were observed in NSCLC patients with local recurrence compared to local recurrence-free patients. This trend did almost reach statistical significance (p = 0.06). For this reason the power of S-MK as a prognostic marker was evaluated in more detail. All NSCLC patients were subdivided in S-MK low (S-MK < 6.71 log pg/ml, < 821 pg/ml) and high expressors (S-MK ≥ 6.71 log pg/ml, ≥ 821 pg/ml). Local recurrence-free survival duration was compared between these two groups using Kaplan–Meier analysis (Fig. 6). In patients with high levels of S-MK local recurrence occurred on average 13 months earlier (20 months post-diagnosis) compared to patients expressing low levels of S-MK (33 months post-diagnosis) in a statistically significant manner (p < 0.03).

**Figure 7:**
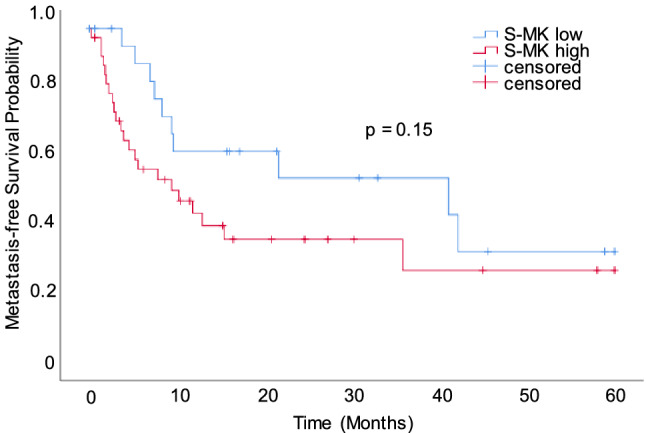
Trend towards later occurrence of metastases in NSCLC patients with low S-MK compared to high S-MK expression. Abbreviations: *S-MK* serum-midkine, *NSCLC* non-small lung cancer. Kaplan–Meier metastasis-free survival analysis of NSCLC patients with low (S-MK < 6.71 log pg/ml; n = 22) and high S-MK levels (S-MK ≥ 6.71 log pg/ml; n = 37). Patients were followed up for 5-years post-surgery. Statistical difference was determined using the log-rank test (p = 0.15).

Further, there was a trend towards distant metastasis occurring earlier in NSCLC patients with high S-MK expression (23 months) versus low S-MK expression (33 months) (Fig. 7), too. However, statistical significance was not quite reached (p = 0.15).

**Figure 8:**
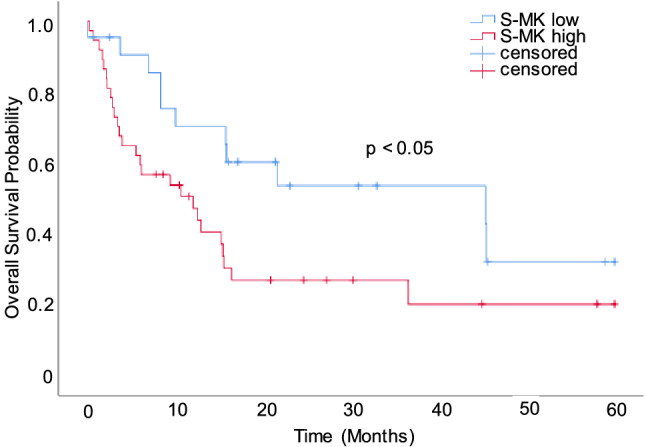
NSCLC patients with low S-MK expression show a longer overall survival than patients with high S-MK expression. Abbreviations: *S-MK* serum-midkine, *NSCLC* non-small lung cancer. Kaplan–Meier curve comparing overall survival of NSCLC patients with low (S-MK < 6.71 log pg/ml; n = 22) and high S-MK levels (S-MK ≥ 6.71 log pg/ml; n = 37). Patients were followed up for 5-years post-surgery. Statistical difference was determined using the log-rank test (p < 0.05).

In a similar manner overall survival duration was compared between NSCLC patients with high and low S-MK expression. Patients with low levels of S-MK (34 months) lived on average 14 months longer than patients with high S-MK levels (20 months) (p < 0.05) as illustrated in Fig. 8. The prognostic value of S-MK was further assessed using cox’s proportional hazards model (Table 2). Here, S-MK was identified as independent prognostic marker (p < 0.048). For patients with S-MK levels > 6.71 log pg/ml the relative risk of death within 5 years was 2.6.

**Table 2:** Prognostic factors for overall survival in NSCLC.

Parameter	Hazard ratio	p-value
S-MK > 6.71 log pg/ml	2.3	0.048
Tumor stage (T)	2.1	0.001
Lymph node status (N)	0.8	0.6
Distant metastasis (M)	1.5	0.6
Residual tumor (R)	2.8	0.08
Grading (G)	3.1	0.009

Multivariate analysis of predictive factors for overall survival of NSCLC patients (n = 59). Statistical significance was determined using Cox regression p < 0.05.

*NSCLC* non-small lung cancer, *S-MK* serum midkine.

## Discussion

Despite advances in various diagnostic technologies, screening tools to detect NSCLC at an early stage are still very limited^17^. So far, conventional biomarkers such as CEA and CYFRA21-1 lack sufficient discriminatory power for reliable screening^18^. Routine CT-scans have been proposed for high-risk patient groups, however, they are associated with radiation exposure to patients and cause a great financial burden on the health system^17,19^. Clearly, early diagnosis is elementary to improve the outcome of NSCLC patients by providing therapy at the earliest possible cancer stage. Thus, innovative, non-invasive screening tools are urgently needed. Furthermore, preoperative biomarkers may predict prognosis and tumor response. Thereby, the best treatment approach can be tailored individually for each NSCLC patient. One such emerging biomarker is the multifunctional cytokine S-MK. Due to overexpression in diverse malignant tumors including NSCLC, it has recently been implicated in the role of tumor biology^6^. Moreover, various reports have suggested a correlation of S-MK expression levels and worse prognosis as well as chemotherapy resistance^11,20–22^.

In this study the value of S-MK both as a diagnostic and as a prognostic marker of NSCLC was assessed retrospectively. This was done by analyzing preoperative S-MK concentration in NSCLS patients. A normal reference range of S-MK expression in healthy individuals has not been established, yet^6^. This is mainly due to the lack of large-scale population studies. For this reason, blood donors served as healthy control in this trial. In line with previous reports^11,18,21^ we found S-MK to be significantly overexpressed in NSCLC patients with a more than threefold median value compared to healthy controls. There was no difference between squamous cell cancer and adenocarcinoma, suggesting a potential functional role of MK in NSCLC regardless of its histological subtype.

To evaluate the clinical utility of S-MK as a screening tool a ROC analysis was carried out. Thereby, a high diagnostic power was found for S-MK to detect NSCLC. So far, a threshold for S-MK levels to differentiate healthy and cancer patients has not been established, yet. Yuan et al. determined an optimal cut-off value of 400 pg/ml to distinguish between malignant and benign pulmonary disease^11^. A cut-off value of 323 pg/ml was proposed by Meng et al. to discriminate malignant from benign thyroid nodules^23^. Based on our data, a threshold of 416 pg/ml S-MK provided optimal sensitivity of 92% and still reasonable specificity of 68%. The results of this study highlight the potential of MK as a clinical biomarker. They suggest that determination of serum levels may aid to diagnose NSCLC pre-surgically.

In order to assess the prognostic value of MK expression in NSCLC patients, preoperative S-MK levels were correlated with clinicopathological parameters. There was a trend of rising S-MK levels with progression of tumor stage and local recurrence. Dividing NSCLS patients into low- and high-grade expressors a statistically significant difference in overall survival and local tumor recurrence was observed. Patients with high levels of S-MK were found to suffer earlier from local recurrence. There was a similar trend for distant cancer recurrence, although not in a statistically significant manner. Moreover, the S-MK level was identified as an independent prediction factor for overall survival. Patients expressing high levels of S-MK had a shorter overall survival and a relative risk of death of 2.6 compared to low expressing patients. Altogether, this prognostic function makes S-MK valuable for personalized outcome prediction in NSCLC patients.

The results discussed above already strongly suggest a certain functional role of MK in the tumor biology of NSCLC. In line with this, previous in vitro studies have confirmed that S-MK is actively involved in tumorigenesis by promoting tumor cell growth, migration and metastasis. MK was found to prevent autophagy-mediated cell death by the Akt/ mTORC1 pathway^24,25^ and exhibit angiogenic activities^26,27^. Moreover, Zhang et al. were able to show that MK enhances chemoresistance by increasing anti-apoptotic protein expression^28^. Furthermore, first studies have demonstrated that tumor growth can effectively be suppressed by S-MK inhibition using antibody trapping^29,30^. Due to these protumorigenic effects, MK may be considered as a novel target for NSCLC treatment approaches in the future.

Further research is required to study the dynamic changes of S-MK level over the course of tumor disease in more detail. In hepatocellular carcinoma and breast cancer it has already confirmed that S-MK decreases after sufficient surgical resection and in turn rebounds in case of incipient relapse^12,13^. This aspect is of great importance when looking at the use of S-MK for postoperative monitoring to predict treatment response as well as disease recurrence.

Limitations of this study are the fact that only healthy controls were compared with NSCLC patients. Benign pulmonary inflammatory nodules are an important differential diagnosis for suspected lung cancer. Therefore, sufficient distinction of NSCLC patients not only from healthy individuals but also from patients with inflammatory disease is necessary. Since MK is a multifunctional growth factor it is known to be slightly increased in inflammation, too. However, the results of previous studies by Xia et al. have already shown that patients with benign pulmonary disease exhibit only marginally elevated S-MK levels lacking a significant difference when compared to healthy controls. On the other hand, there was a significant overexpression of S-MK in NSCLC patients compared to patients with benign pulmonary disease. Thus, it can be postulated that the slight increase in benign pulmonary disease is different from the overexpression in lung cancer and can be neglected.

Although S-MK was confirmed to be significantly overexpressed In NSCLC patients, its sensitivity and specificity is not yet sufficient for it to be applied as a sole screening marker for NSCLC in clinical routine. This is partly due to the low incidence of NSCLC in the general population. Having mentioned other biomarkers such as CYFRA21 and CEA, S-MK may, however, contribute to the discriminatory power when used as an additional biomarker. Therefore, it would be worth to reassess the diagnostic value of MK using a multimarker panel in future studies.

In conclusion, this monocentric study has confirmed that MK is significantly overexpressed in patients with NSCLC and that S-MK can serve as an indicator for patient outcomes. Overall, our data weighs in favor for the minimally-invasive S-MK as an additional diagnostic and prognostic marker for NSCLC which could be of great relevance to clinical practice and therefore warrants further research.
